# Gradient-Based Curvature-Only Time-Derivative Coupling for Surface Hopping and Coherent Switching with Decay of Mixing: Application to SO_2_ and Molecular Tully Models

**DOI:** 10.1021/acs.jctc.6c01219

**Published:** 2026-09-10

**Authors:** Feven-Alemu Korsaye, Leticia González

**Affiliations:** Institute of Theoretical Chemistry, Faculty of Chemistry, 27258 University of Vienna, Währinger Straße 17, 1090 Vienna, Austria

## Abstract

Time-derivative couplings (TDCs) are central ingredients in mixed quantum–classical simulations of nonadiabatic dynamics, yet their evaluation remains a key practical challenge when analytic nonadiabatic coupling vectors or wave function overlaps are unavailable. Curvature-driven approximations provide an appealing alternative by evaluating TDCs from adiabatic potential energies or gradients along nuclear trajectories, motivated by the one-dimensional Baeck–An model. In this work, we examine curvature-based TDC approximations within trajectory surface hopping (TSH) and coherent switching with decay of mixing (CSDM), and introduce a modified gradient-based curvature-only formulation that retains the directional-curvature contribution to the TDC while omitting the explicit acceleration-dependent term at all times in the coupling evaluation. The performance of energy-based, gradient-based, and curvature-only TDCs is evaluated for four benchmark systems of increasing dynamical complexity: ethylene, DMABN, fulvene, and SO_2_. For ethylene, all curvature-driven schemes closely reproduce reference dynamics obtained with analytic nonadiabatic couplings. For DMABN and fulvene, which involve repeated nonadiabatic passages and back-transfer, the curvature-only approximation yields improved agreement with reference populations by reducing overpopulation effects observed with standard curvature formulations. In the multistate singlet–triplet dynamics of SO_2_, standard curvature-based TDCs introduce spurious population transfer to the ground state, an artifact that is significantly decreased by the curvature-only scheme in both TSH and CSDM. Overall, the results show that removing the acceleration-dependent contribution leads to a more robust curvature-driven TDC approximation across a wide range of nonadiabatic regimes, while retaining the conceptual simplicity and efficiency of curvature-based approximations.

## Graphical abstract

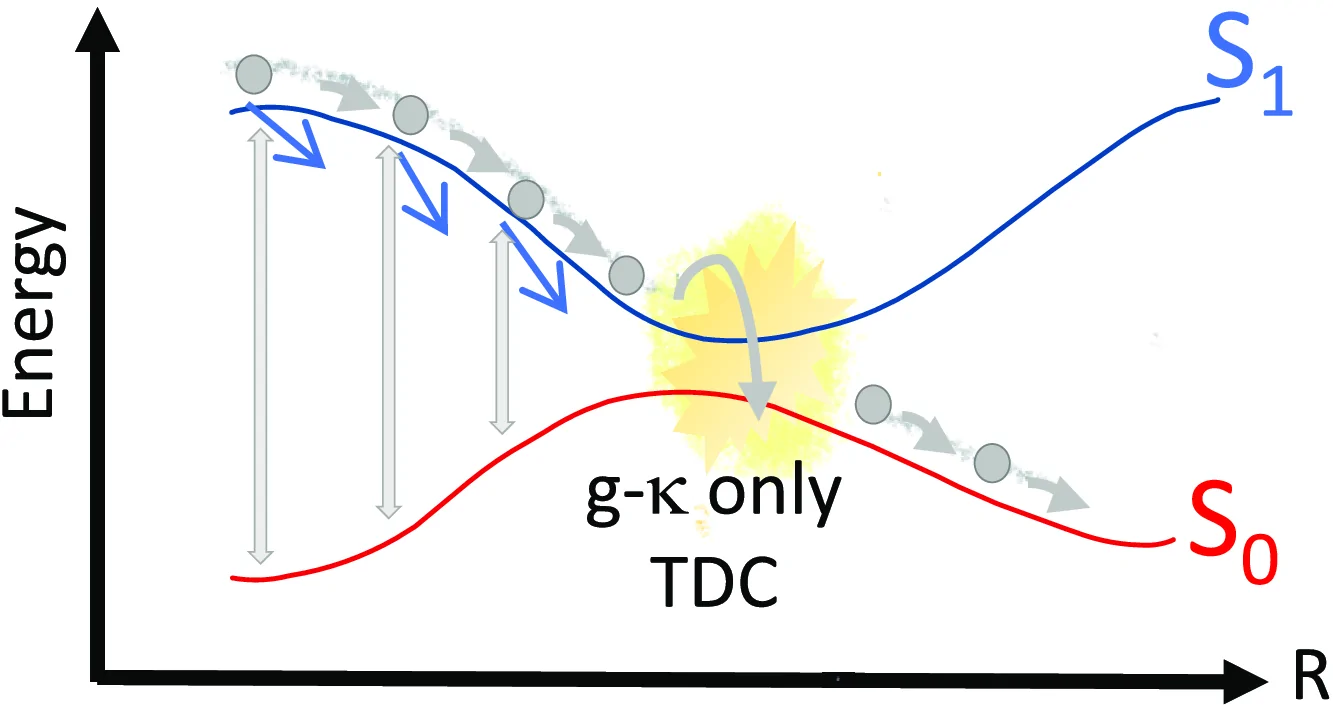

## 1 Introduction

Mixed quantum–classical trajectory methods, such as trajectory surface hopping (TSH)^1^ and coherent switching with decay of mixing (CSDM),^2,3^ provide a practical route to simulate nonadiabatic processes in full-dimensional molecular systems.^4–9^ In the adiabatic representation, the electronic amplitudes are propagated using time-derivative couplings (TDCs), which quantify the instantaneous rate at which nuclear motion transfers amplitude between adiabatic electronic states and can be evaluated as the nuclear velocity projection of the nonadiabatic coupling (NAC) vectors. Obtaining these couplings can be a significant bottleneck in direct dynamics simulations, as analytic NAC vectors are unavailable for many electronic-structure methods, and even when available, their evaluation is computationally demanding. Overlap-based approaches, like the local diabatization^10^ scheme are popular,^11^ as they bypass the explicit calculation of NAC vectors by using wavefunction overlaps between successive time steps. They are however not directly applicable when electronic wave functions are not available, as is typically the case for machine-learning-based potentials. Curvature-driven time-derivative coupling approximations (κTDC),^12^ also referred to as time-dependent Baeck–An couplings,^13^ address this limitation by estimating TDCs from adiabatic potential information (gradients and/or energies) along the nuclear trajectory. In these approaches, the coupling magnitude is related to the curvature of the adiabatic energy gap along the trajectory time coordinate, motivated by the one-dimensional Baeck–An expression for the strength of nonadiabatic coupling near an avoided-crossing.^14^

Two practical variants can be used to evaluate the gap curvature entering κTDC: an *energy-based* form obtained from doubly numerical second derivative of the energy gap and a *gradient-based* form obtained from finite differences of the projected gap slope. For sufficiently small nuclear time steps, both discretizations are expected to converge to the same continuous-time limit. In practice, the gradient-based form is often numerically more stable, since it relies on lower-order finite differences of energies. The energy-based form can nevertheless be computationally attractive in TSH simulations when gradients are needed only for the active state, whereas the gradient-based form requires gradients for all states included in the dynamics. For self-consistent-potential methods (e.g., CSDM-type dynamics),^2,3^ all state gradients are required in any case, and the gradient-based form becomes the natural choice. In both the energy- and gradient-based κTDC variants, the scalar curvature-driven TDC can be used to construct an effective NAC vector.^12^ This effective vector can then provide a coupling direction when required, e.g., for velocity adjustment after successful hops in TSH and for defining the nonadiabatic force contribution in self-consistent-potential dynamics.

Because curvature-driven schemes require only local potential-energy information, they are applicable in dynamical settings where analytic NAC vectors or wave function overlaps are not available. In recent benchmarking and application studies, curvature-based time-based TDCs have been applied to a wide range of nonadiabatic dynamics problems,^15–25^ including applications where explicit NACs or wave function overlaps are impractical, such as dynamics driven by machine-learned potentials.^26–30^ At the same time, curvature-driven schemes can be challenged by dynamics involving more than two strongly coupled states, by trivial crossings, and by small discontinuities in energies or gradients.^25,31,32^ Such discontinuities are especially relevant in multireference electronic-structure calculations, where they may arise from changes in active-space orbital character or state ordering, and curvature-based approximations, that rely on numerical derivatives, can amplify these artifacts.^31^ Consequently, reliable applications of curvature-driven approaches typically require reasonably continuous potential energies, and, when discontinuities occur, additional safeguards external to the coupling formula.^13^

In the present work, we introduce a modified discretization within the gradient-based curvature-driven framework. The modified curvature-only TDC approximation (κ-onlyTDC), defined in detail below, retains only the directional-curvature component of the energy-gap curvature at all times in the coupling estimate. This simplification is motivated by the need to reduce sensitivity to short-time numerical fluctuations and algorithmic velocity adjustments (e.g., momentum rescaling) that can introduce coupling features that are not directly tied to the local curvature of the adiabatic energy gap.

The performance of the κ-only approximation is evaluated on two complementary benchmark sets. First, we employ the molecular Tully models^33^—ethylene, 4-dimethylaminobenzonitrile (DMABN), and fulvene—which reproduce, in full dimensionality key features of Tully’s standard one-dimensional test scenarios.^1^ Specifically, ethylene provides a dynamics dominated by a single nonadiabatic event, DMABN probes repeated nonadiabatic passages via multiple crossings, and fulvene exhibits reflection and recrossing following passage through a sloped conical intersection. These models provide full-dimensional tests in which the nonadiabatic dynamics is dominated by well-characterized avoided-crossing or conical-intersection regions, allowing the sensitivity to the coupling approximation to be assessed in a controlled setting. In a posterior step, we consider SO_2_ as an example of the system where excited-state dynamics involves strong vibronic coupling within the low-lying singlet manifold and competing intersystem crossing to nearby three triplet states.^34,35^ This combination of vibronic and spin–orbit-mediated couplings provides a challenging test for curvature-based coupling approximations beyond the purely singlet, two-state avoided-crossing scenarios represented by the molecular Tully models. For each of these benchmark systems, we compare five different treatments of the time-derivative couplings within TSH and CSDM: (i) energy-based κTDC, (ii) gradient-based κTDC, (iii) gradient-based κ-onlyTDC, (iv) analytic NAC-vector couplings (where available), and (v) an overlap-based local diabatization scheme. This set of comparisons is designed to isolate the effect of using a scalar TDC approximation in electronic propagation from other algorithmic differences.

## 2 Methodology

### 2.1 κTDC

In the adiabatic electronic representation, the NAC vector between adiabatic states {ϕ_*I*_} is defined as

1 $$d_{\mathrm{IJ}}\left(R\right)=\left\langle \phi_{I}\left(R\right)\left|\nabla_{R}\right|\phi_{J}\left(R\right)\right\rangle$$

and the corresponding TDC required to propagate the electronic amplitudes is given by

2 $$\sigma_{\mathrm{IJ}}\left(t\right)=\left\langle \phi_{I}\left(R\left(t\right)\right)\left|\frac{d}{dt}\right|\phi_{J}\left(R\left(t\right)\right)\right\rangle =Ṙ\left(t\right)\cdot d_{\mathrm{IJ}}\left(R\left(t\right)\right)$$

The curvature-based approximation is motivated by the one-dimensional Baeck–An expression for the magnitude of the NAC along a nuclear coordinate *q* ^14^

3 $$\left|d_{\mathrm{IJ}}^{\mathrm{BA}}\left(q\right)\right|\approx \frac{1}{2}{\left(\frac{1}{\Delta V_{\mathrm{IJ}}\left(q\right)}\frac{d^{2}}{dq^{2}}\Delta V_{\mathrm{IJ}}\left(q\right)\right)}^{1/2}$$

where Δ*V*_*IJ*_(*q*) = *V_I_* (*q*) – *V*_*J*_(*q*) > 0 is the local adiabatic energy gap, with *I* > *J* the locally upper and lower adiabatic states, respectively. A time-domain, along-the-trajectory generalization of the 1D Baeck–An curvature model, using the trajectory parameter *t* as the effective 1D coordinate yields the curvature-approximated TDC (κTDC)^12,13^

4 $$\sigma_{\mathrm{IJ}}^{\kappa }\left(t\right)\approx \frac{1}{2}{\left(\frac{1}{\Delta V_{\mathrm{IJ}}\left(t\right)}\frac{d^{2}}{dt^{2}}\Delta V_{\mathrm{IJ}}\left(t\right)\right)}^{1/2},I>J$$

with antisymmetry enforced by σ_*JI*_^κ^ = −σ_*IJ*_^κ^. Since negative values of the square-root argument are expected to occur mainly away from locally avoided crossings, where the nonadiabatic coupling is small, the corresponding coupling element is set to zero.

To clarify the relation between the coordinate-curvature form in eq 3 and the time-domain curvature in eq 4, let us consider one-dimensional motion along an effective coordinate *q*(*t*). Along the trajectory, Δ*V*_*IJ*_(*t*) = Δ*V*_*IJ*_(*q*(*t*)) and the chain rule yields

5 $$\frac{d\Delta V_{\mathrm{IJ}}}{dt}=\mathrm{q̇}\frac{d\Delta V_{\mathrm{IJ}}}{dq}$$

and

6 $$\frac{d^{2}\Delta V_{\mathrm{IJ}}}{dt^{2}}={\mathrm{q̇}}^{2}\frac{d^{2}\Delta V_{\mathrm{IJ}}}{dq^{2}}+\mathrm{q̈}\frac{d\Delta V_{\mathrm{IJ}}}{dq}$$

Equation 6 separates the time curvature of the gap into a *directional-curvature* contribution, proportional to the coordinate curvature d^2^Δ*V*_*IJ*_/d*q* ^2^, and an *acceleration-weighted slope* contribution, proportional to dΔ*V*_*IJ*_/d*q*. In the avoided-crossing regime motivating eq 3, the relevant portion of the trajectory is centered near a minimum of the gap along the effective coordinate, for which dΔ*V*_*IJ*_/d*q* is small. Under this condition, the second term in eq 6 becomes negligible, and one obtains the approximate mapping

7 $$\frac{d^{2}\Delta V_{\mathrm{IJ}}}{dq^{2}}\approx \frac{1}{{\mathrm{q̇}}^{2}}\frac{d^{2}\Delta V_{\mathrm{IJ}}}{dt^{2}}$$

Substituting eq 7 into the Baeck–An estimate (eq 3) and rearranging gives the Baeck–An approximation for the TDC (eq 4).

In practice, the second time derivative of the energy gap in eq 4 can be evaluated either from energies (energy-based κTDC) or from gradients and velocities (gradient-based κTDC). In the energy-based variant, d^2^Δ*V*_*IJ*_/d*t* ^2^ is approximated by backward finite differences of Δ*V*_*IJ*_(*t*). For example, using a three-point backward approximation

8 $${\frac{d^{2}\Delta V_{\mathrm{IJ}}}{dt^{2}}|}_{t}\approx \frac{\Delta V_{\mathrm{IJ}}\left(t\right)-2\Delta V_{\mathrm{IJ}}\left(t-\Delta t\right)+\Delta V_{\mathrm{IJ}}\left(t-2\Delta t\right)}{\Delta t^{2}}$$

and, once sufficient history is available, a higher-order four-point backward approximation may be used

9 $${\frac{d^{2}\Delta V_{\mathrm{IJ}}}{dt^{2}}|}_{t}\approx \frac{2\Delta V_{\mathrm{IJ}}\left(t\right)-5\Delta V_{\mathrm{IJ}}\left(t-\Delta t\right)+4\Delta V_{\mathrm{IJ}}\left(t-2\Delta t\right)-\Delta V_{\mathrm{IJ}}\left(t-3\Delta t\right)}{\Delta t^{2}}$$

Alternatively, d^2^Δ*V*_*IJ*_/d*t* ^2^ can be evaluated in a gradient-based form by rewriting it as the time derivative of the gap slope along the trajectory

10 $$\frac{d^{2}}{dt^{2}}\Delta V_{\mathrm{IJ}}\left(t\right)=\frac{d}{dt}\left[\nabla_{R}\Delta V_{\mathrm{IJ}}\left(t\right)\cdot Ṙ\left(t\right)\right]$$

which is approximated by backward finite differences as

11 $${\frac{d^{2}}{dt^{2}}\Delta V_{\mathrm{IJ}}|}_{t+\Delta t}\approx \frac{\nabla_{R}\Delta V_{\mathrm{IJ}}\left(t+\Delta t\right)\cdot Ṙ\left(t+\Delta t\right)-\nabla_{R}\Delta V_{\mathrm{IJ}}\left(t\right)\cdot Ṙ\left(t\right)}{\Delta t}$$

Equations 8, 9 and 11 converge to the same continuous-time limit as Δ*t* → 0, but they differ in numerical behavior and in the electronic-structure information required at each time step.

### 2.2 κ-OnlyTDC

The κTDC definition in eq 4 is based on the time curvature of the gap, d^2^Δ*V*_*IJ*_/d*t* ^2^, which can be rewritten as the time derivative of the gap slope along the trajectory, eq 10. Expanding this derivative yields the exact decomposition

12 $$\frac{d}{dt}\left[\nabla_{R}\Delta V_{\mathrm{IJ}}\cdot Ṙ\right]=\underset{\mathrm{directional‐curvature}\;\mathrm{contribution}}{\underset{︸}{\left(\frac{d}{dt}\nabla_{R}\;\Delta V_{\mathrm{IJ}}\;\right)\cdot \overset{\;}{Ṙ}}}+\underset{\mathrm{acceleration‐weighted}\;\mathrm{slope}\;\mathrm{contribution}}{\underset{︸}{\nabla_{R}\;\Delta V_{\mathrm{IJ}}\;\cdot \mathrm{R̈}\;}}$$

Thus, both energy and gradient-based κTDC implicitly incorporates both the directional-curvature and acceleration-weighted slope contributions in eq 12. As discussed above, in the avoided-crossing regime motivating the Baeck–An model, the acceleration-weighted slope contribution is often negligible, and the time-curvature estimate behaves consistently with the Baeck–An TDC. Outside such regimes, for sufficiently small Δ*t*, smooth trajectories imply small Δ**Ṙ**, however, in practice, they can undergo abrupt changes due to numerical fluctuations or algorithmic operations (e.g., due to momentum rescaling after hops in TSH), which appear as large discrete accelerations over a single time step. Because κTDC relies on numerical time derivatives, such impulsive contributions can be amplified and may introduce spurious coupling away from strong coupling regions.

Motivated by this observation, we define a *curvature-only* (κ-only) variant by retaining the local directional curvature of the adiabatic gap along the trajectory and omitting the acceleration-weighted slope term in eq 12 *at all times in the coupling estimate*, i.e., not only in crossing regions

13 $$\frac{d^{2}}{dt^{2}}\Delta V_{\mathrm{IJ}}\left(t\right)\approx \left(\frac{d}{dt}\nabla_{R}\Delta V_{\mathrm{IJ}}\left(t\right)\right)\cdot Ṙ\left(t\right)$$

By substitution into eq 4, this yields

14 $$\sigma_{\mathrm{IJ}}^{\kappa ‐\mathrm{only}}\left(t\right)\approx \frac{1}{2}{\left[\frac{1}{\Delta V_{\mathrm{IJ}}\left(t\right)}\left(\frac{d}{dt}\nabla_{R}\Delta V_{\mathrm{IJ}}\left(t\right)\right)\cdot Ṙ\left(t\right)\right]}^{1/2}$$

As for the standard κTDC, antisymmetry is enforced as σ_*JI*_^κ‑only^ = −σ_*IJ*_^κ‑only^, and negative square-root arguments are set to zero.

In practice, the directional-curvature contribution in eq 14 can be evaluated by a backward finite difference of the gradient difference and a time-centered projection of the nuclear velocity over the interval [*t*, *t* + Δ*t*]­

15 $$\left(\frac{d}{dt}\nabla_{R}\Delta V_{\mathrm{IJ}}\right)\cdot {Ṙ|}_{t+\Delta t}\approx \frac{\nabla_{R}\Delta V_{\mathrm{IJ}}\left(t+\Delta t\right)-\nabla_{R}\Delta V_{\mathrm{IJ}}\left(t\right)}{\Delta t}\cdot \frac{Ṙ\left(t+\Delta t\right)+Ṙ\left(t\right)}{2}$$

This gradient-based form is the natural implementation of eq 14, since it directly discretizes the time derivative of the gap-gradient difference along the trajectory. An analogous energy-only implementation is less direct because energies sampled as a function of time do not provide a strict separation of the two contributions in eq 12.

### 2.3 Effective NAC Vector *G*_*IJ*_

In addition to a scalar time-derivative coupling for the electronic propagation, nonadiabatic coupling vectors are required for defining nonadiabatic force contributions in self-consistent-potential dynamics. In the context of curvature-driven CSDM, an effective NAC vector has been formulated from the scalar time-derivative coupling used in the electronic propagation.^12^ The construction restricts the effective coupling direction to the subspace spanned by the difference-gradient vector **g**_*IJ*_(*t*) = ∇_**R**_ΔV_*IJ*_(*t*) and the nuclear velocity **Ṙ**(*t*). Accordingly, the effective NAC vector is expressed as

16 $$G_{\mathrm{IJ}}\left(t\right)=g_{\mathrm{IJ}}\left(t\right)+\alpha_{\mathrm{IJ}}\left(t\right)Ṙ\left(t\right)$$

where α_*IJ*_(*t*) is chosen such that the contraction of **G**_*IJ*_(*t*) with the nuclear velocity reproduces the scalar coupling

17 $$Ṙ\left(t\right)\cdot G_{\mathrm{IJ}}\left(t\right)=\sigma_{\mathrm{IJ}}\left(t\right)$$

Solving eq 17 for α_*IJ*_(*t*) gives

18 $$\alpha_{\mathrm{IJ}}\left(t\right)=\frac{\sigma_{\mathrm{IJ}}\left(t\right)-Ṙ\left(t\right)\cdot g_{\mathrm{IJ}}\left(t\right)}{Ṙ\left(t\right)\cdot Ṙ\left(t\right)}$$

In order to conserve the total nuclear angular momentum and the position of the center of mass, the effective coupling vector **G**_*IJ*_(*t*) is projected to remove the six-dimensional subspace corresponding to overall translation and rigid-body rotation.^36^ The resulting projected effective NAC vector **G**_*IJ*_^*Q*^(*t*) is used in CSDM to define the coupling direction entering the nonadiabatic force contribution within the self-consistent potential. In addition, the same vector can also be used when a coupling direction is required for velocity adjustment in TSH simulations.

In the present work, σ_*IJ*_(*t*) in eq 18 is provided either by the curvature-approximated coupling σ_*IJ*_^κ^(*t*) or by the curvature-only coupling σ_*IJ*_^κ‑only^(*t*). Thus, the construction of **G**_*IJ*_(*t*) is identical for κ and κ-only; only the scalar input σ_*IJ*_(*t*) differs.

## 3 Computational Details

### 3.1 Electronic Structure

Since the chemical systems serve only as models to illustrate the capabilities of the new methods rather than to achieve quantitative accuracy, the electronic-structure method underlying the dynamical simulations is deliberately chosen as simple as possible while capturing qualitative correctness. Accordingly, the potential-energy surfaces and gradients of ethylene relied on the SA(3)-CASSCF­(2,2)/6-31G* level of theory,^37–40^ averaging over three states with two active electrons in two active π/π* orbitals. For DMABN, energies and gradients were also computed on-the-fly but using linear-response TDDFT^41,42^ within the Tamm–Dancoff approximation^43^ and the LC-PBE^44^ functional with 6-31G basis set. Fulvene was treated with SA(3)-CASSCF­(6,6)/6-31G* level of theory, with the active space including six electrons distributed over three pairs of π/π* molecular orbitals. All CASSCF calculations were carried out with the OpenMolcas v23 ^45^ quantum chemistry package, and TDDFT calculations were done with the Orca 6.0 ^46^ software. For SO_2_, dynamics was simulated on pre-calculated potential-energy surfaces that used a linear vibronic coupling^47^ (LVC) model at the MR-CIS­(6,6)/ANO-RCC-VDZP level of theory,^48,49^ taken from ref 50.

### 3.2 Nonadiabatic Dynamics

Nonadiabatic dynamics simulations were performed using TSH and CSDM. For each dynamics method, we compared five interstate couplings treatments: (i) energy-based κTDC (e-κTDC), (ii) gradient-based κTDC (g-κTDC), (iii) gradient-based κ-onlyTDC (g-κ-onlyTDC), (iv) TDC obtained from analytic NACs (except for DMABN treated with TDDFT), and (v) the local diabatization scheme to propagate the electronic amplitudes without explicit couplings. When employing local diabatization for the electronic propagation within CSDM, coupling vectors were still required to evaluate the nuclear equations of motion; in this case, we employed effective NAC vectors constructed from the κ-only approximation.

Initial conditions for all trajectory ensembles were stochastically sampled from a ground-state vibrational Wigner distribution.^51^ Each trajectory was then started by vertically exciting the molecule to an excited electronic state appropriate for the photochemical process under study. Ethylene and fulvene trajectories were initiated in the first excited singlet state at the Franck–Condon geometry, and the two singlet S_0_ and S_1_ states were included in the nonadiabatic dynamics. In DMABN, the initial excitation was prepared in the bright locally excited S_2_ state and the three lowest-lying singlet states (S_0_–S_2_) were included in the dynamics. For SO_2_, the trajectories were started in the optically allowed S_1_ state and the dynamics were performed including 3 singlet and 3 triplet states. A total of 200 trajectories were propagated for ethylene and fulvene, 100 trajectories for DMABN, and 120 trajectories for SO_2_. Nuclear motion was propagated using the velocity-Verlet^52^ algorithm with fixed nuclear time steps of 0.1 fs for ethylene, fulvene, and SO_2_, and 0.5 fs for DMABN. A total number of 200 substeps were used for the integration of the electronic equation of motion. Trajectories were propagated for 80 fs in ethylene, 190 fs in DMABN, 40 fs in fulvene, and 700 fs in SO_2_.

In all TSH simulations, nuclear velocities were adjusted uniformly scaling the full nuclear velocity vector after successful hops to enforce total-energy conservation. This choice was adopted as a uniform protocol across all coupling treatments to isolate the impact of the TDC approximation on the electronic propagation and hopping statistics in TSH, avoiding additional variability associated with defining a coupling direction from approximate NAC vectors. Additionally, the energy-based decoherence correction^53^ was applied to account for electronic decoherence. All nonadiabatic molecular dynamics simulations were performed in the diagonal representation using the SHARC4.0 ^54^ molecular dynamics package, and κ-onlyTDC approximation has been implemented into a local development version of SHARC4.0.

## 4 Results and Discussion

In this section, we report and analyze nonadiabatic dynamics simulations performed using TSH and CSDM for the four benchmark systems: ethylene, DMABN, fulvene, and SO_2_, spanning progressively to more demanding nonadiabatic regimes. Population dynamics are reported as quantum populations, and the internal consistency of the simulations was verified by comparison with classical populations, which are provided in the Supporting Information. For ethylene and fulvene, results obtained using analytic NACs are taken as the reference. For DMABN, analytic NACs are not available within the employed TDDFT framework, we therefore compare against ab initio multiple spawning (AIMS) populations reported in ref 55 under the same electronic-structure setup. For all reference population curves, statistical uncertainty is estimated using a two-sided 95% confidence interval

19 $$\Delta p\left(t\right)=\pm 1.96\sqrt{\frac{p\left(t\right)\left[1-p\left(t\right)\right]}{N_{traj}}}$$

where *p*(*t*) is the population at time *t* and *N*_traj_ is the number of trajectories. For SO_2_, we consider as reference the multiconfiguration time-dependent Hartree (MCTDH)^56^ simulations of ref 50 obtained with the same LVC Hamiltonian as used here.

### 4.1 Ethylene

Ethylene is a compact benchmark for testing coupling approximations in a two-state regime. Following π → π* excitation, its early-time photodynamics is dominated by rapid S_1_ → S_0_ relaxation through a well-characterized nonadiabatic region associated with torsion and pyramidalization around the C=C bond.^57^ Owing to its small size and the presence of a single dominant nonadiabatic pathway involving two adiabatic states, ethylene has been extensively studied using both trajectory-based and wave packet-based approaches.^3,58–63^ Because the relaxation is dominated by a single, relatively localized nonadiabatic event in a two-state manifold, ethylene provides a favorable setting for curvature-based TDC approximations, as it will be demonstrate below.

Figure 1 shows the time evolution of the S_1_ population for ethylene obtained with TSH (panel a) and CSDM (panel b). In both cases, the results obtained with analytic NACs show a S_1_ population decay, reaching approximately 30% at 80 fs. The results obtained with local diabatization and all curvature-based TDC closely follow the reference NAC-based dynamics over most of the simulation time. Small quantitative deviations emerge at longer times, where both g-κ and e-κ exhibit a small overpopulation of S_1_ during the final ∼10 fs of the simulations, amounting to approximately a 10% difference relative to the NAC reference. The g-κ-only and LD description remain in closer agreement with the NAC-based population decay up to the end of the propagation window. This behavior is consistent across both TSH and CSDM and suggests that, for ethylene, the removal of the acceleration-dependent contribution at all times does not degrade the quality of the curvature-driven estimate and it slightly improves robustness at longer times. Overall, for ethylene, both energy- and gradient-based curvature TDCs provide a reliable description of the S_1_ population decay in TSH and CSDM. The κ-only variant retains this level of agreement while exhibiting slightly improved stability at longer times.

**Figure 1:**
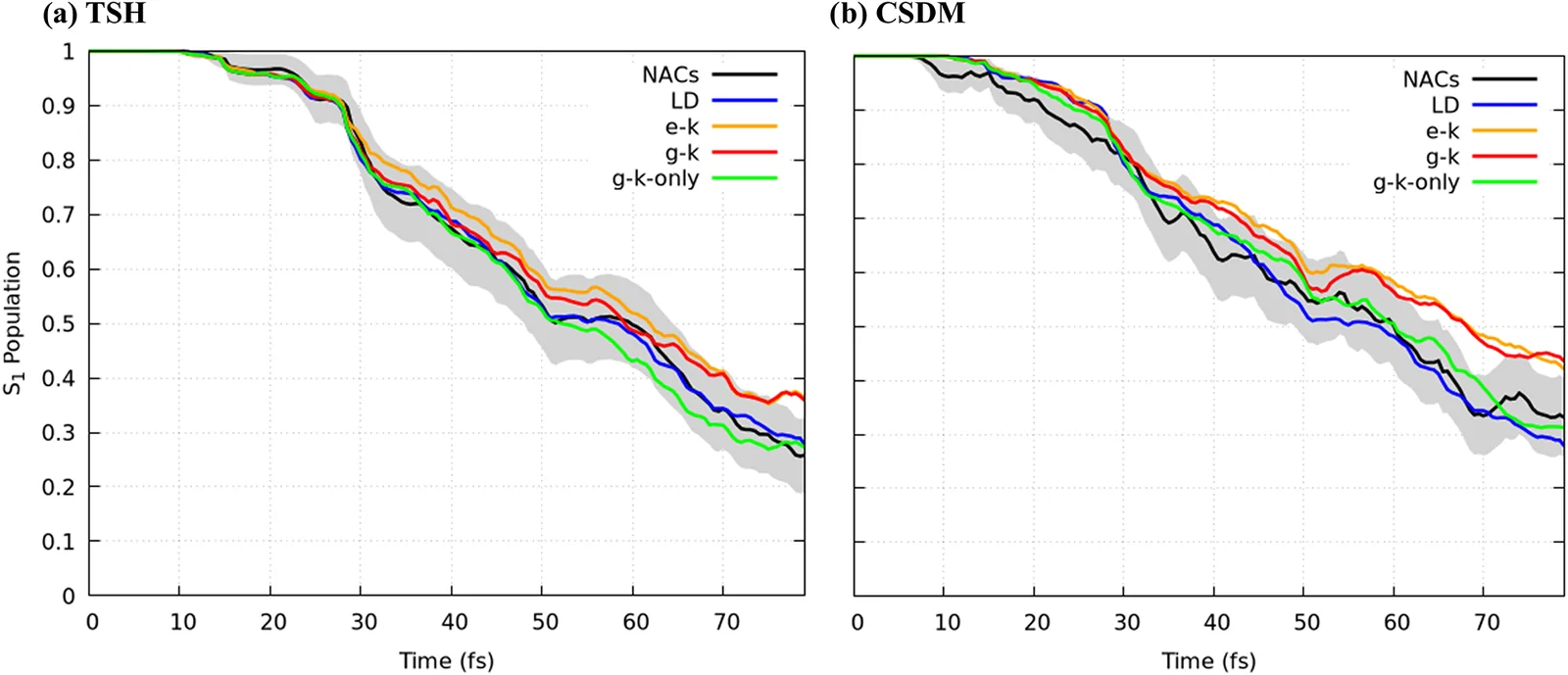
Time evolution of the adiabatic S_1_ population in ethylene obtained with TSH (a) and CSDM (b) using analytic nonadiabatic couplings (NACs, in black), local diabatization (LD, in blue), e-κ (orange), g-κ (red), and g-κ-only (green).

### 4.2 DMABN

DMABN has been used to benchmark nonadiabatic dynamics methods because its excited-state relaxation involves multiple nonadiabatic events and extended population exchange between the involved electronic states.^33,55,64–68^ Following photoexcitation to the bright S_2_ state, the system undergoes rapid internal conversion to the S_1_ state, followed by slower population redistribution. This dynamics is characterized by repeated passages through regions of moderate nonadiabatic coupling rather than a single localized event, making DMABN a more stringent test than ethylene to probe the ability of approximate coupling schemes to capture both early-time transfer and longer-time population redistribution. Indeed, the performance of curvature-based TDC approximations for DMABN is expected to differ from that observed for ethylene; while curvature-driven schemes rely on the local curvature of the adiabatic energy gap along the nuclear trajectory, the presence of multiple nonadiabatic encounters and extended coupling regions in DMABN increases the sensitivity to how the curvature is evaluated.

Figure 2 reports the time evolution of the S_2_ population for DMABN obtained with TSH (panel a) and CSDM (panel b) compared against AIMS populations. In both propagation schemes, all coupling treatments predict an ultrafast initial decay of the S_2_ population within the first ∼10 fs, with the population dropping to approximately 40%. This early-time behavior is largely insensitive to the choice of coupling approximation and reflects the strong initial nonadiabatic coupling out of the Franck–Condon region. Clear differences among the coupling schemes emerge at later times. In both TSH and CSDM, the local diabatization and curvature-only populations closely follow the AIMS reference during ∼130 fs, with the S_2_ population dropping below 20% by approximately 40–45 fs. In contrast, both the gradient-based and energy-based curvature schemes predict a higher S_2_ population at intermediate and long times, oscillating between approximately 15 and 35% and thus overestimating the population of S_2_ state. This trend is consistent in both TSH and CSDM dynamics.

**Figure 2:**
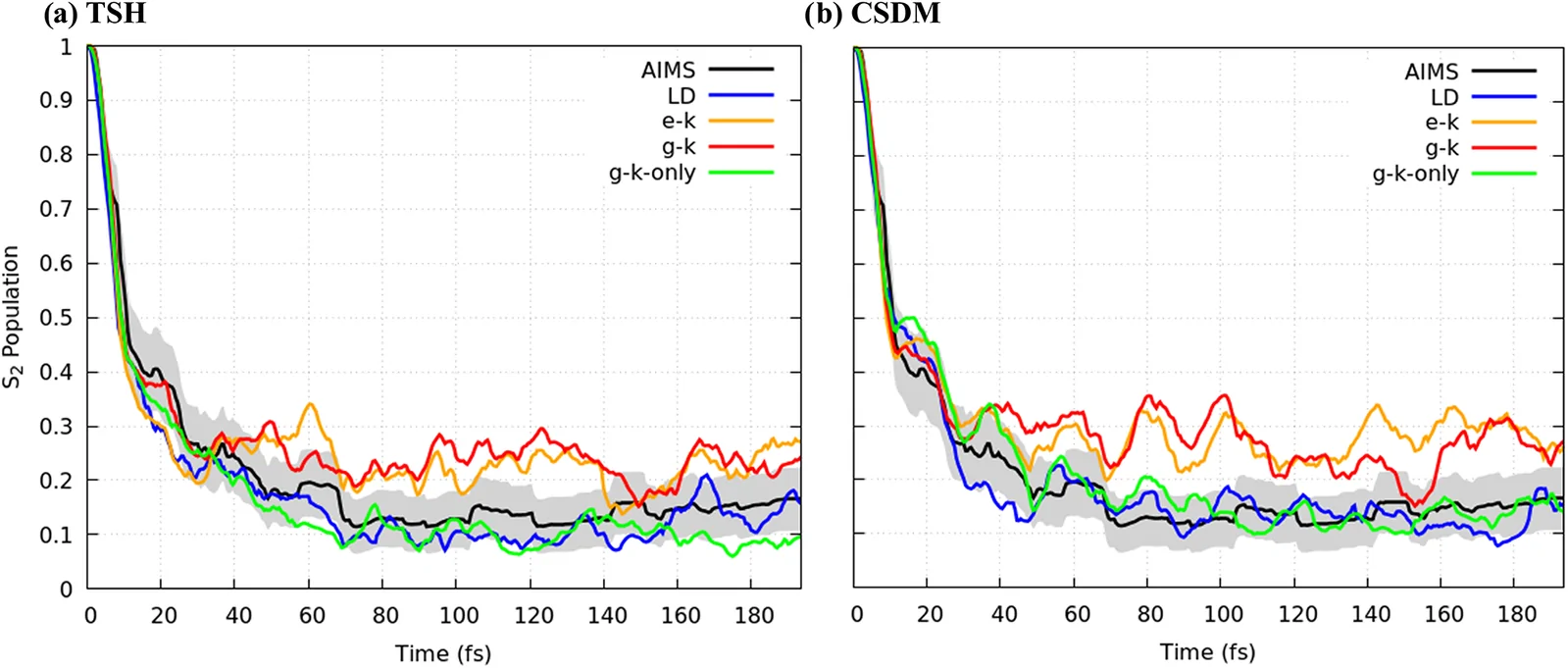
Time evolution of the adiabatic S_2_ population in DMABN obtained with TSH (a) and CSDM (b) using local diabatization (LD, in blue), e-κ (orange), g-κ (red), and g-κ-only (green). AIMS dynamics (black) is used as reference.

To further characterize the systematic overpopulation of S_2_ observed with g-κ and e-κ, Table 1 reports the number of interstate transitions between S_2_ and S_1_. In both dynamic frameworks, the standard curvature formulations (e-κ and g-κ) exhibit substantially larger number of transitions than g-κ-only and also exceed the corresponding LD counts. For TSH, the total number of S_1_ ↔ S_2_ transitions is 654 and 618 for e-κ and g-κ, respectively, compared with 487 for LD and 330 for g-κ-only. A similar trend is observed in CSDM, with 568 and 641 transitions for e-κ and g-κ, respectively, compared with 478 for LD and 344 pointer-state switches for g-κ-only. The time-binned transition analysis reported in the Supporting Information shows that the excess transition activity is not confined to a single time interval. During the initial 0–30 fs period, which is dominated by S_2_ → S_1_ relaxation, the number of S_1_ → S_2_ transitions is similar among the curvature treatments. After this initial period, e-κ and g-κ consistently exhibit more S_1_ → S_2_ transitions than g-κ-only over several successive time windows, while the excess in S_2_ → S_1_ transitions is already present in the first interval and persists throughout the subsequent dynamics. These results show that e-κ and g-κ produce more frequent back-and-forth transitions between S_2_ and S_1_. The additional transition events indicate enhanced recurrent S_1_ ↔ S_2_ switching with the standard curvature formulations relative to both LD and g-κ-only, accompanying the persistent S_2_ overpopulation observed in the corresponding population curves.

**Table 1:** Number of Accepted Hops and Pointer-State Switches between S_2_ and S_1_ in DMABN for TSH and CSDM

	TSH				CSDM			
transition	LD	e-κ	g-κ	g-κ-only	LD	e-κ	g-κ	g-κ-only
S_1_ → S_2_	203	289	269	128	198	248	286	140
S_2_ → S_1_	284	365	349	202	280	320	355	204

### 4.3 Fulvene

The excited-state relaxation of fulvene is governed by repeated passages through a pronounced S_1_/S_0_ conical-intersection region. This gives rise to a staircase-like decay of the excited-state population, with partial recoveries between successive nonadiabatic passages over a few tens of femtoseconds, making fulvene a popular benchmark for nonadiabatic dynamics and for assessing the sensitivity of TSH simulations to algorithmic choices.^13,23,33,69–72^ Because SH dynamics for fulvene can depend strongly on velocity rescaling^23,69^ due to the frequent back-and-forth transitions between S_1_ and S_0_, here, we use the same velocity-rescaling protocol across all coupling treatments to isolate the effect of the TDC approximation and to enable a direct comparison to the analytic NAC-based dynamics.

Figure 3 reports the time evolution of the S_1_ population for fulvene obtained with TSH (panel a) and CSDM (panel b). In both cases, all methods predict a rapid decay of the S_1_ population within the first ∼12 fs, transferring approximately 70–80% of the population to S_0_. Subsequently, a partial back-transfer to S_1_ is observed, followed by a second encounter with the coupling region around ∼24 fs that again reduces the S_1_ population. A second repopulation of S_1_ occurs after ∼30 fs, reflecting the repeated exploration of the conical-intersection seam.

**Figure 3:**
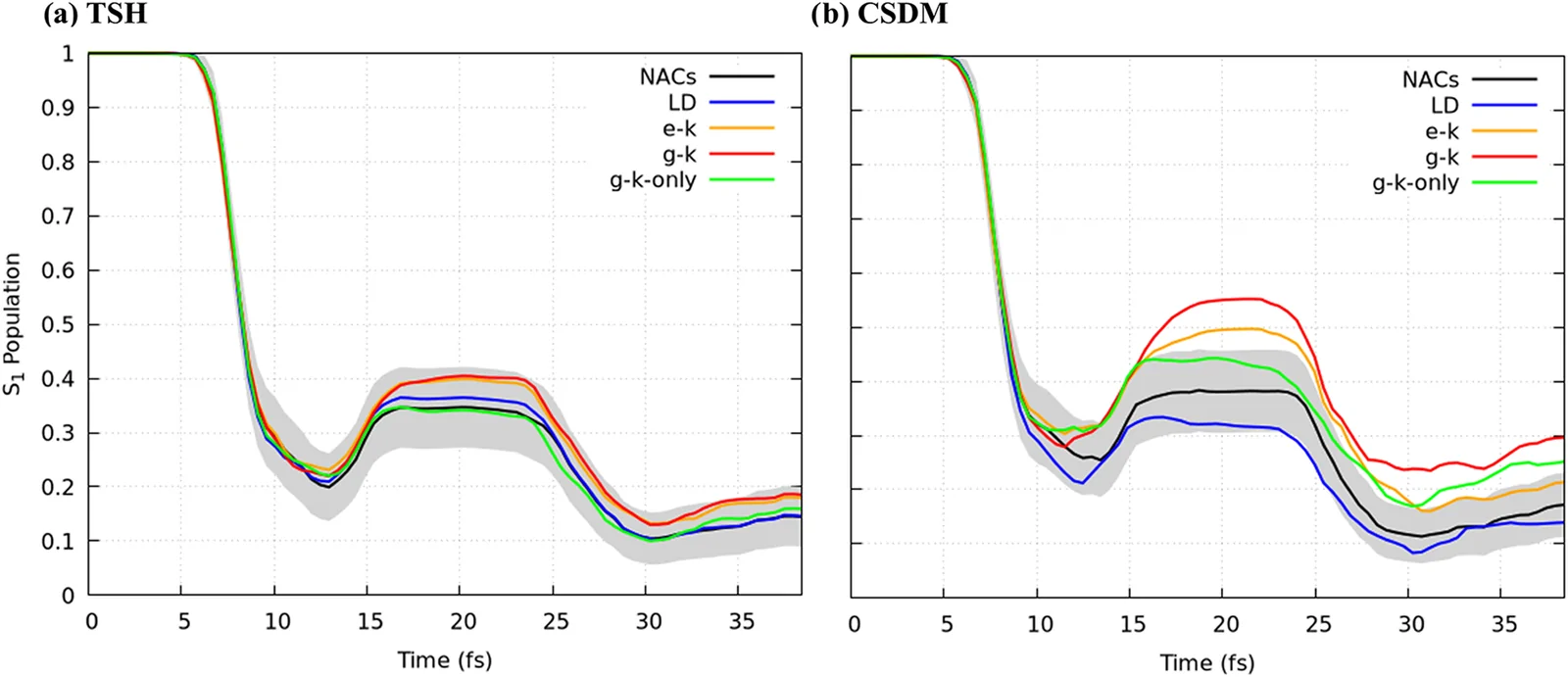
Time evolution of the adiabatic S_1_ population in fulvene obtained with TSH (a) and CSDM (b) using analytic NACs (black), local diabatization (LD, in blue), e-κ (orange), g-κ (red), and g-κ-only (green).

While all simulations capture the qualitative sequence of decay and back-transfer, there are some quantitative differences. In TSH dynamics, analytic NACs predict a rapid depopulation of S_1_ by approximately 80% at ∼12 fs, followed by back-transfer in the 15–24 fs interval, after which about 33% of the population remains in S_1_. Among the curvature-based schemes, both g-κ and e-κ agree well with the analytic NAC reference; however, g-κ-only reproduces the reference most closely, yielding an S_1_ population of approximately 33%. After the second decay event near ∼24 fs, g-κ-only again provides the closest quantitative agreement, whereas g-κ and e-κ predict slightly higher S_1_ populations. Qualitatively similar trend is observed in the CSDM simulations, although larger deviations are present for all curvature-based coupling treatments. In CSDM, such deviations are amplified because effective NAC vectors—constructed from the scalar curvature-based TDCs—enter the nuclear equations of motion through the self-consistent potential. The restriction of the effective NAC to the two-dimensional subspace spanned by the nuclear velocity and the gap-gradient vector introduces an additional approximation, and the errors in the scalar coupling evaluation can feed back into the nuclear dynamics and affect the electronic populations relative to those obtained with analytic NACs.

These interpretations are supported by single-trajectory diagnostics. Figure 4 compares the TDCs along representative TSH (panel a) and CSDM (panel b) trajectories that do not undergo back-hopping. While g-κ-only qualitatively reproduces the timing and magnitude of the dominant coupling events observed with analytical NACs, g-κ and e-κ predict very similar coupling profiles and exhibit additional coupling features in regions where the analytical-NAC coupling is negligible. These spurious contributions correlate with enhanced back-transfer and account for the overpopulation observed in the ensemble-averaged population. Similar trends are observed for additional representative trajectories, whose TDC comparisons are reported in the Supporting Information.

**Figure 4:**
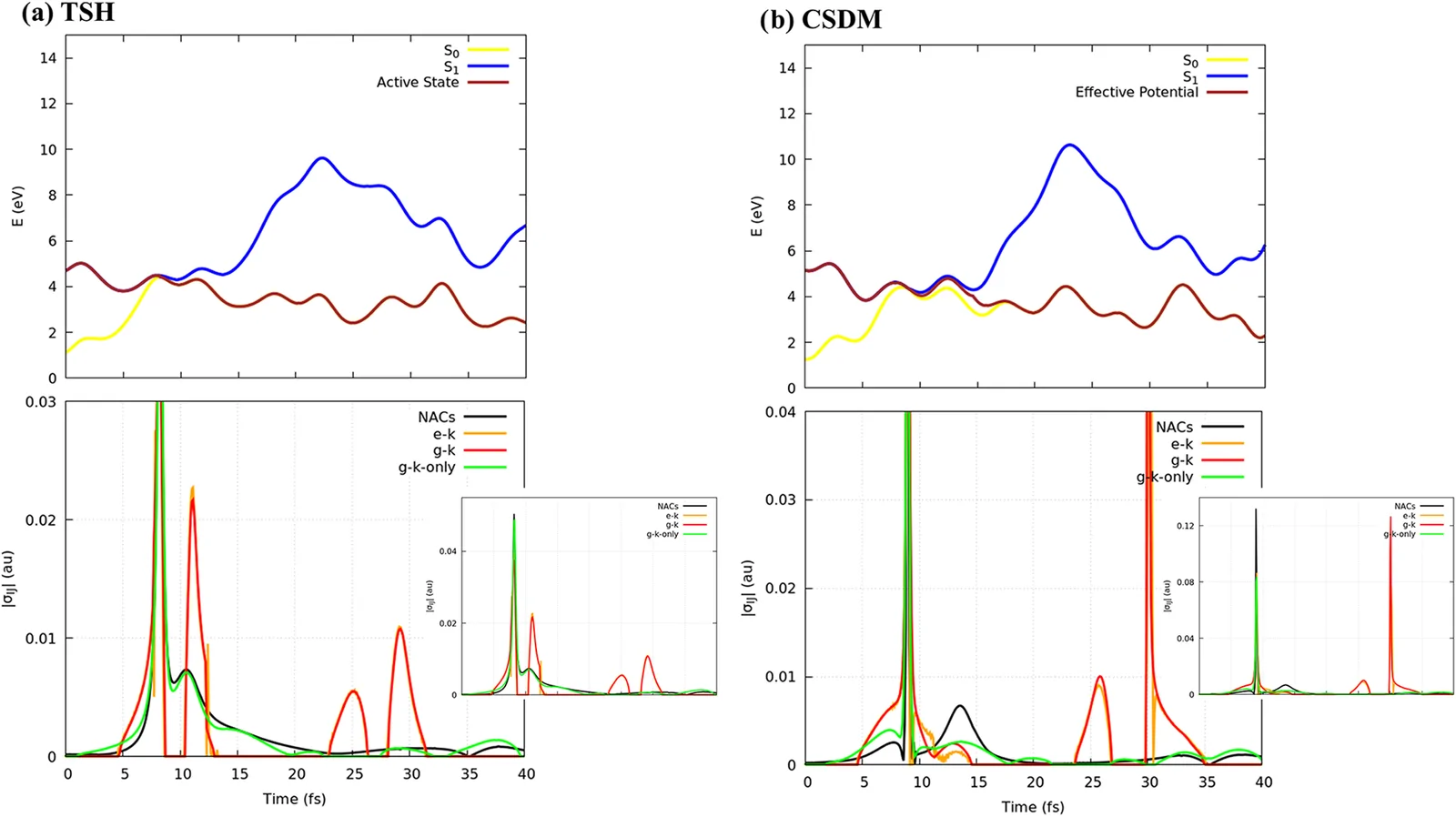
Representative trajectories in fulvene. Top: Adiabatic potential energies of S_0_ (yellow) and S_1_ (blue), together with the active-state potential (brown) for TSH (a) or self-consistent effective potential in CSDM (b). Bottom: Corresponding magnitude of the time derivative coupling |σ_01_(*t*)| obtained from analytic NACs (black) and from curvature-driven schemes (orange: e-κ; red: g-κ; green: g-κ-only).

### 4.4 SO_2_

Sulfur dioxide represents a simple but challenging benchmark for nonadiabatic dynamics. Following photoexcitation in the near-UV, the dynamics of SO_2_ is governed by strong vibronic coupling among the lowest singlet states and by efficient intersystem crossing, leading to a rapid population redistribution between singlet and triplet manifolds.^34,35,73^ From the perspective of curvature-driven approximations, SO_2_ represents a particularly challenging case. In contrast to the two-state benchmarks discussed above, the dynamics involve multiple near-degenerate states, rapid changes in electronic character, and frequent passages through regions of small adiabatic energy gaps. These challenges are amplified by the presence of both singlet and triplet manifolds and the long simulation times required to resolve population redistribution.

In the present work, SO_2_ dynamics is described using a LVC model parameterized for three singlets (^1^A_1_, ^1^A_2_, ^1^B_1_) and three triplets states (^3^A_2_, ^3^B_1_, ^3^B_2_). This LVC model has been shown to reproduce the essential photodynamics when treated with MCTDH, and prior work has further shown that TSH combined with local diabatization yields diabatic populations in good agreement with the corresponding MCTDH/LVC reference for this system.^50^ The published MCTDH/LVC dynamics is therefore used here as the reference for comparison, and the corresponding population curves are reported in the Supporting Information. Figures 5 and 6 report diabatic populations obtained with TSH and CSDM, respectively, for different coupling treatments, while the corresponding MCH populations are provided in the Supporting Information. Using TSH with local diabatization, the initial excitation populates predominantly the ^1^B_1_ state, following, within the first 10 fs, the electronic character rapidly evolves, and population is transferred predominantly to the ^1^A_2_ diabatic state, which becomes the dominant singlet component at short times. At later times, oscillatory population exchange between ^1^B_1_ and ^1^A_2_ is observed. In parallel, population transfer to the triplet manifold builds up on a sub-picosecond time scale, dominated by ^3^B_2_ state, which reaches ∼0.28 by 700 fs. These main features qualitatively match the reference behavior reported for MCTDH/LVC dynamics.

**Figure 5:**
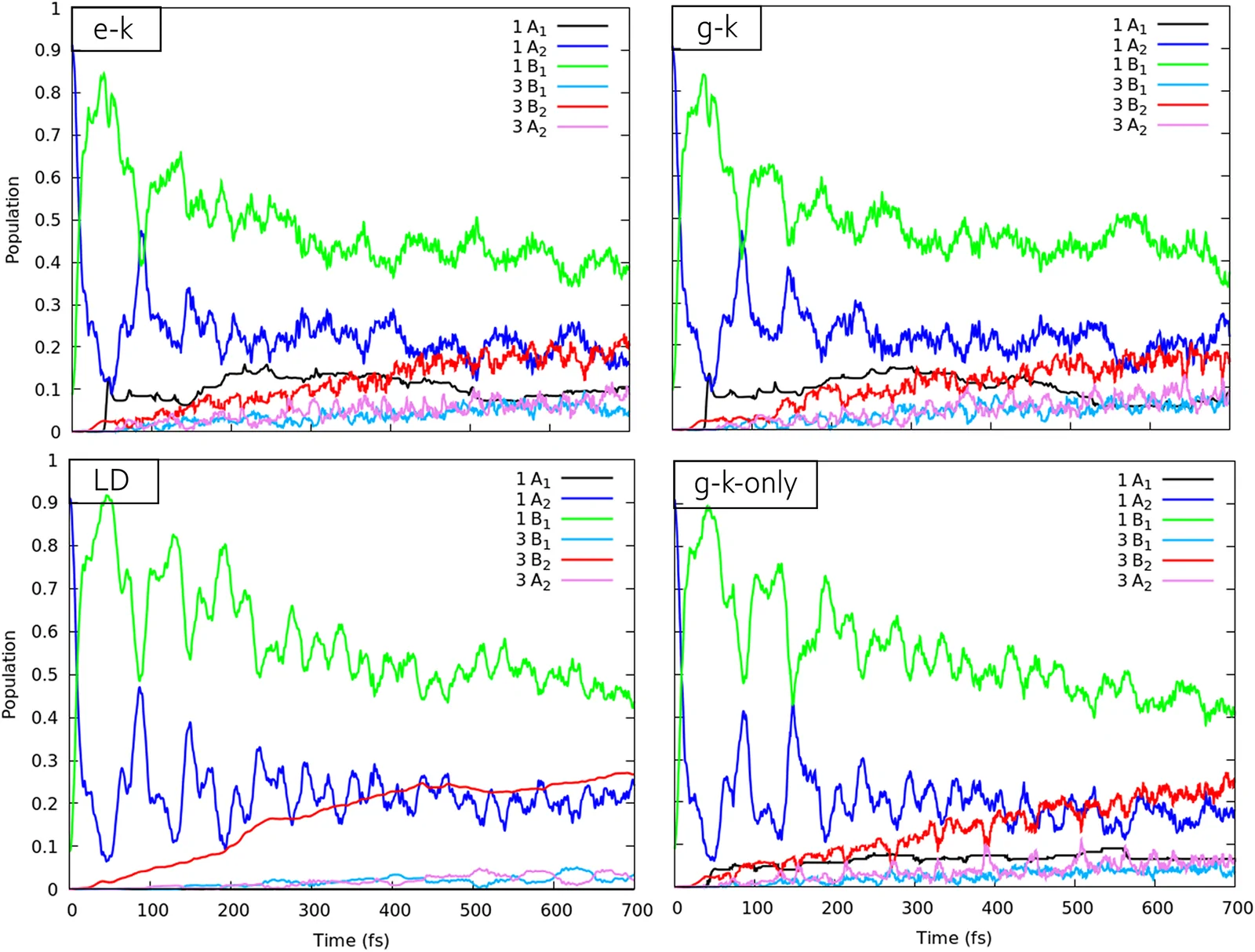
Diabatic state populations for SO_2_ from TSH dynamics, shown for four coupling treatments: e-κ (top left), g-κ (top right), local diabatization (LD, bottom left), and g-κ-only (bottom right). Line colors indicate diabatic states as labeled in each panel.

**Figure 6:**
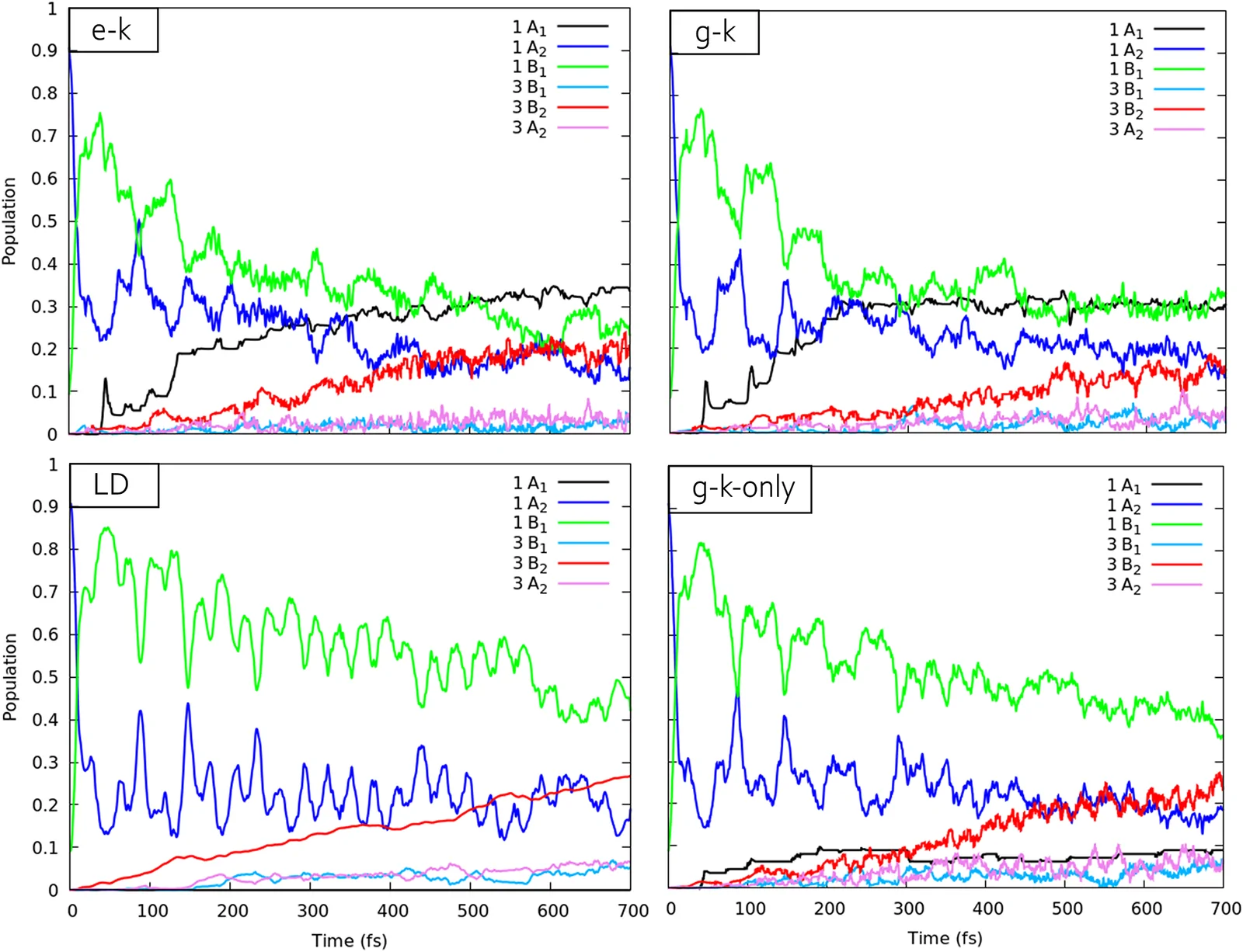
Diabatic state populations for SO_2_ from CSDM dynamics, shown for four coupling treatments: e-κ (top left), g-κ (top right), local diabatization (LD, bottom left), and g-κ-only (bottom right). Line colors indicate diabatic states as labeled in each panel.

When energy-based and gradient-based curvature-driven TDCs are employed within TSH, the main qualitative features of the early-time dynamics are reproduced. In particular, the ultrafast redistribution of population from ^1^B_1_ to ^1^A_2_ within the first 10 fs and the buildup of triplet population are captured. However, both e-κ and g-κ predict an unphysical symmetry-forbidden population transfer to the singlet ground state ^1^A_1_. This artifact appears already at ∼40 fs, with population flowing from ^1^A_2_ and subsequently also from ^1^B_1_ to ^1^A_1_. The presence of this spurious ground-state population indicates that these curvature-based approximations generate non-negligible effective couplings between states that are not directly coupled in the reference dynamics. In CSDM, since the curvature-based TDCs are additionally used to construct effective coupling vectors entering the nuclear propagation and decoherence correction, the errors in the curvature-based TDCs are fed back into the nuclear dynamics and accumulate over time. As a result, the deficiencies of e-κ and g-κ become more pronounced in CSDM simulations, as shown in Figure 6. For both e-κ and g-κ, this feedback leads to a substantial overpopulation of the ^1^A_1_ ground state and to a concomitant distortion of the singlet–triplet branching ratios, leading to a significant deviation from the local diabatization-based CSDM reference. A substantial improvement is instead obtained when using the curvature-only variant g-κ-only. Already at the level of TSH, g-κ-only reduces the unphysical population transfer to ^1^A_1_ and yields singlet and triplet populations that are much closer to the reference. In CSDM, these improvement are even more pronounced, showing more stable effective NAC vectors constructed from g-κ-only TDCs. Indeed, although a small residual ground-state population remains, its magnitude is strongly reduced compared to the e-κ and g-κ results, and the overall redistribution among singlet and triplet states is significantly improved. This trend is also reflected in the number of trajectories that satisfy the same total-energy conservation criterion over the full propagation. For SO_2_, the success rate is higher for g-κ-only than for the full curvature variants in both methods (TSH: 118/120 for g-κ-only vs 89/120 for e-κ and 88/120 for g-κ; CSDM: 107/120 for g-κ-only vs 95/120 for e-κ and 88/120 for g-κ). The pronounced artifacts observed for e-κ and g-κ in SO_2_ can indeed be rationalized by the multistate nature of the dynamics. In regions where several electronic states are close in energy, numerical evaluation of the time curvature of the energy gap can generate large effective couplings even in the absence of strong physical nonadiabatic interactions. The acceleration-dependent contribution retained in the standard curvature formulations is sensitive to such situations and can trigger spurious transitions. By construction, the g-κ-only approximation removes this contribution, thereby reducing sensitivity to rapid velocity changes and to numerical noise in multistate crossing regions. The remaining deviations from the reference are plausibly related to the intrinsic limitations of curvature-based couplings, which depend solely on local energy curvature and do not encode wavefunction-based information on state mixing.

Finally, we see that local diabatization-based electronic propagation yields the most stable population dynamics for SO_2_ in both TSH and CSDM. In dense multistate regions, the overlap-based propagation is comparatively robust, whereas curvature-driven couplings—constructed from numerical time derivatives of energy gaps—can generate spurious transitions when several states are simultaneously near-degenerate. The SO_2_ results therefore delineate a regime in which local diabatization should be preferred when wavefunction information is available, while curvature-based approximations are better suited as surrogate couplings when such information is inaccessible.

## 5 Conclusions

Curvature-driven approximations offer a practical approach to estimate time-derivative couplings in nonadiabatic dynamics when analytic nonadiabatic coupling vectors or wave function overlaps are unavailable. Building on the Baeck–An motivation, we have examined energy-based and gradient-based curvature formulations within trajectory surface hopping (TSH) and coherent switching with decay of mixing (CSDM), and introduced a gradient-based curvature-only variant that explicitly removes the acceleration-dependent contribution to the time curvature of the adiabatic energy gap at all times in the coupling evaluation.

Across four benchmark systems spanning progressively more demanding nonadiabatic regimes, distinct performance trends emerged. For ethylene, a two-state system dominated by a single localized coupling event, all curvature-driven formulations closely reproduce reference dynamics obtained with analytic nonadiabatic couplings with only minor numerical differences at long times. In DMABN and fulvene, where the dynamics repeated passages through coupling-active regions and pronounced back-transfer, standard curvature-based time-derivative couplings overestimate excited-state populations after the initial ultrafast decay. In these systems, the curvature-only approximation yields improved agreement with reference populations, indicating that the acceleration-dependent contribution can bias the effective time-integrated coupling when less-localized nonadiabatic regions appear. The most stringent test is provided by SO_2_, where coupled singlet and triplet manifolds give rise to a dense multistate dynamics. In this regime, curvature-based time-derivative couplings activate spurious coupling channels and lead to unphysical population transfer to the singlet ground state, particularly when used within CSDM where curvature-derived couplings also influence nuclear propagation. The curvature-only variant reduces these artifacts and provides the most robust population dynamics among the curvature-based schemes considered here. Beyond the electronic propagation, the effective nonadiabatic coupling vectors constructed from curvature-only time-derivative couplings may be used as a practical coupling direction for momentum rescaling in TSH when analytic couplings are not available.

These benchmarks highlight a regime in which curvature-only time derivative couplings can provide an improved description by retaining the directional-curvature contribution while reducing sensitivity to numerical noise and algorithmic velocity change without introducing additional empirical parameters or increasing computational cost. Although this simplification generally leads to a qualitative improvement, curvature-based approximations are constructed only from local potential-energy information and do not incorporate state-specific electronic information, and therefore, they retain intrinsic limitations that become more pronounced in dense multistate manifolds.

## Supplementary Material



## Data Availability

Input and output files from the nonadiabatic dynamics simulations of the Tully molecular models and SO_2_ are available on Zenodo at DOI: 10.5281/zenodo.20717339. The scripts and code used to generate and analyze the results are publicly available at: 10.5281/zenodo.20593204, SHARC release 4.0.^54^

## Notes

Classical populations of ethylene, DMABN, fulvene, and SO_2_ obtained with TSH and CSDM. Single-trajectory time-derivative coupling for representative fulvene trajectories, comparing analytic NAC-based TDCs with e-κ, g-κ, and g-κ-only approximations (PDF)
