# Supplementary material for: Gradient-Based Curvature-Only Time-Derivative Coupling for Surface Hopping and Coherent Switching with Decay of Mixing: Application to SO2 and Molecular Tully Models
Source: J Chem Theory Comput. 2026 Sep 10;22(18):9290–301. doi: 10.1021/acs.jctc.6c01219 (PMC13602848; doi:10.1021/acs.jctc.6c01219)
Supplement: Supplementary file 1 [file ct6c01219_si_001.pdf]

Supporting Information: Gradient-based Curvature-only Time-derivative Coupling for Surface Hopping and Coherent Switching with Decay of Mixing: Application to SO_2 and Molecular Tully Models

Feven-Alemu Korsaye*, Leticia González*

Institute of Theoretical Chemistry, Faculty of Chemistry, University of Vienna, Währinger Straße 17, 1090 Vienna, Austria

E-mail: feven.alemu.korsaye@univie.ac.at;leticia.gonzalez@univie.ac.at

Contents

1 Classical Populations S-3

2 Number of $S_2 \rightarrow S_1$ and $S_1 \rightarrow S_2$ hops in DMABN S-7

3 Single-trajectory TDCs S-8

4 Reference MCTDH/LVC dynamics for SO_2 S-9

5 MCH population for SO_2 S-10

References S-10

1 Classical Populations

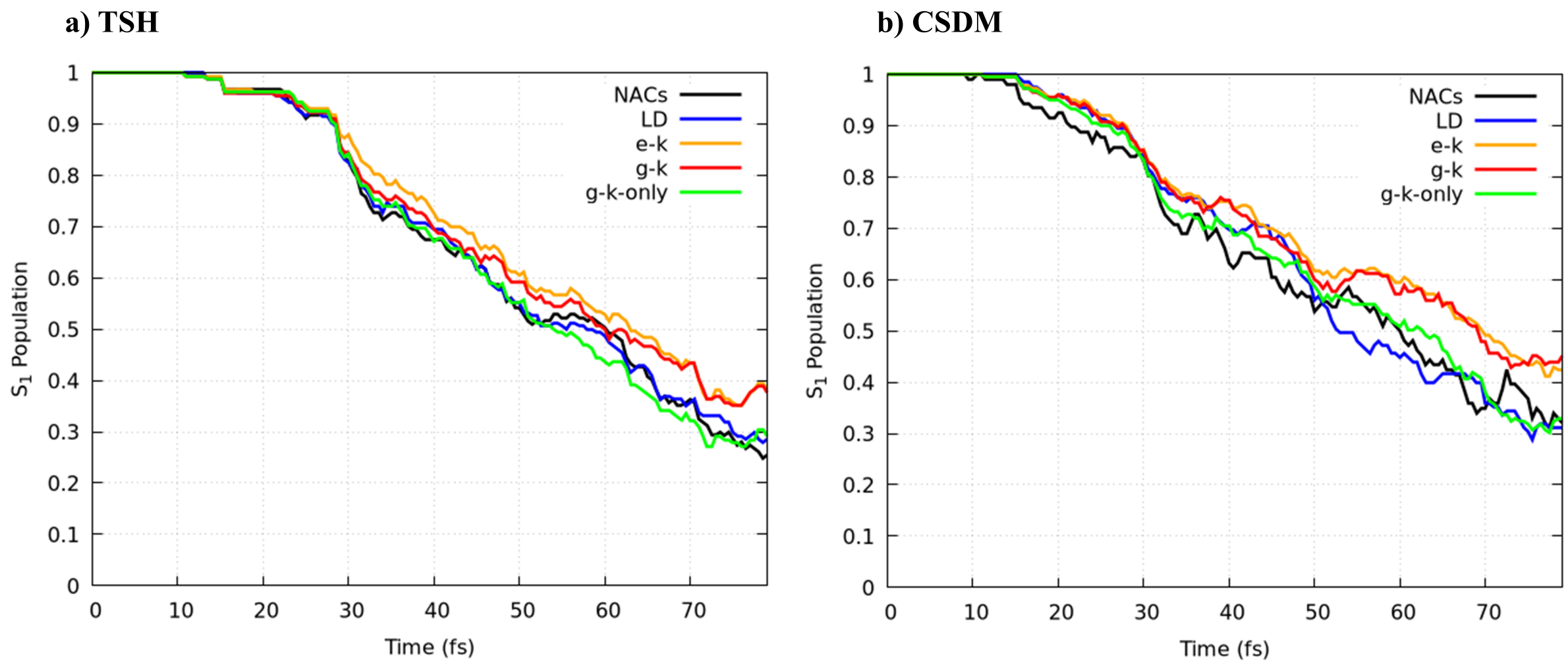

Figure S1: S_1 classical population in ethylene obtained with TSH (a) and CSDM (b) using analytic nonadiabatic couplings (NACs, in black), local diabsatization (LD, in blue), e-κ (orange), g-κ (red) and g-κ-only (green).

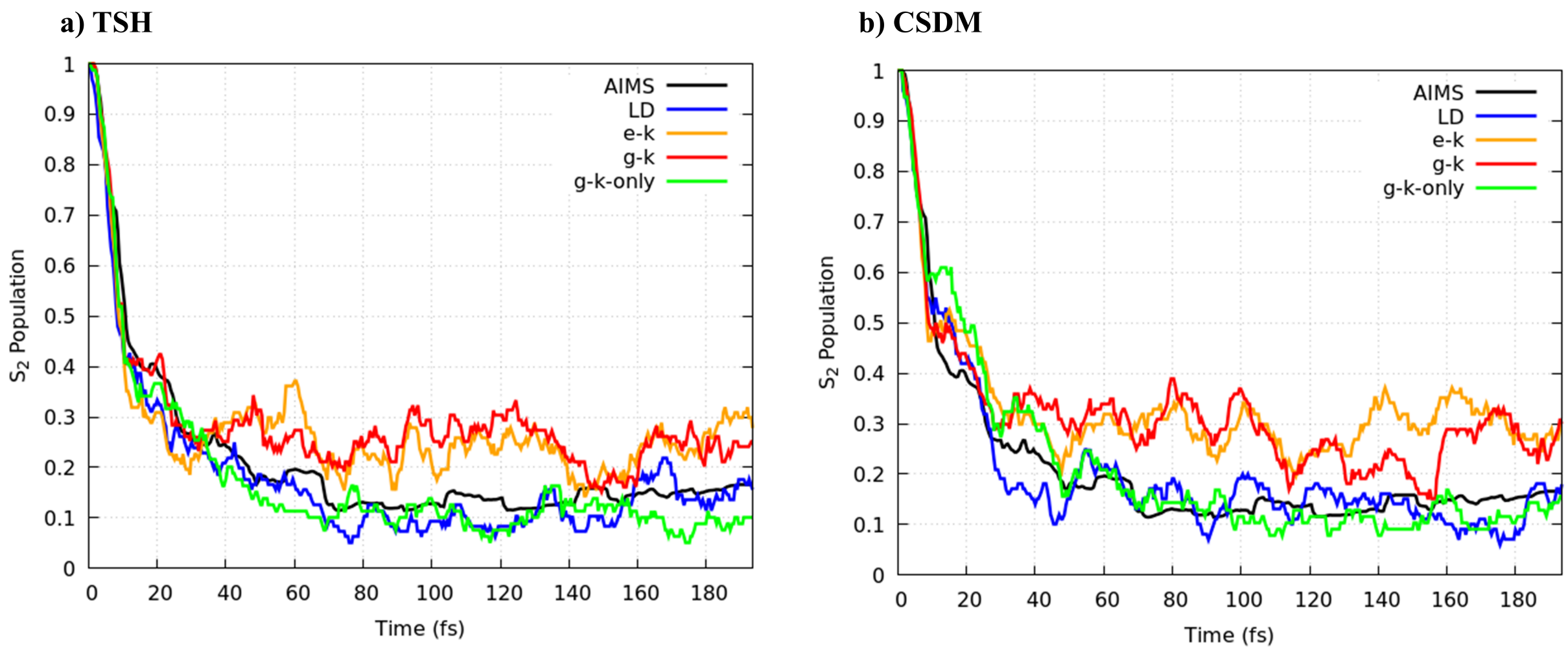

Figure S2: S_2 classical population in DMABN obtained with TSH (a) and CSDM (b) using local diabatization (LD, in blue), e-κ (orange), g-κ (red) and g-κ-only (green). AIMS dynamics (black) is used as reference.

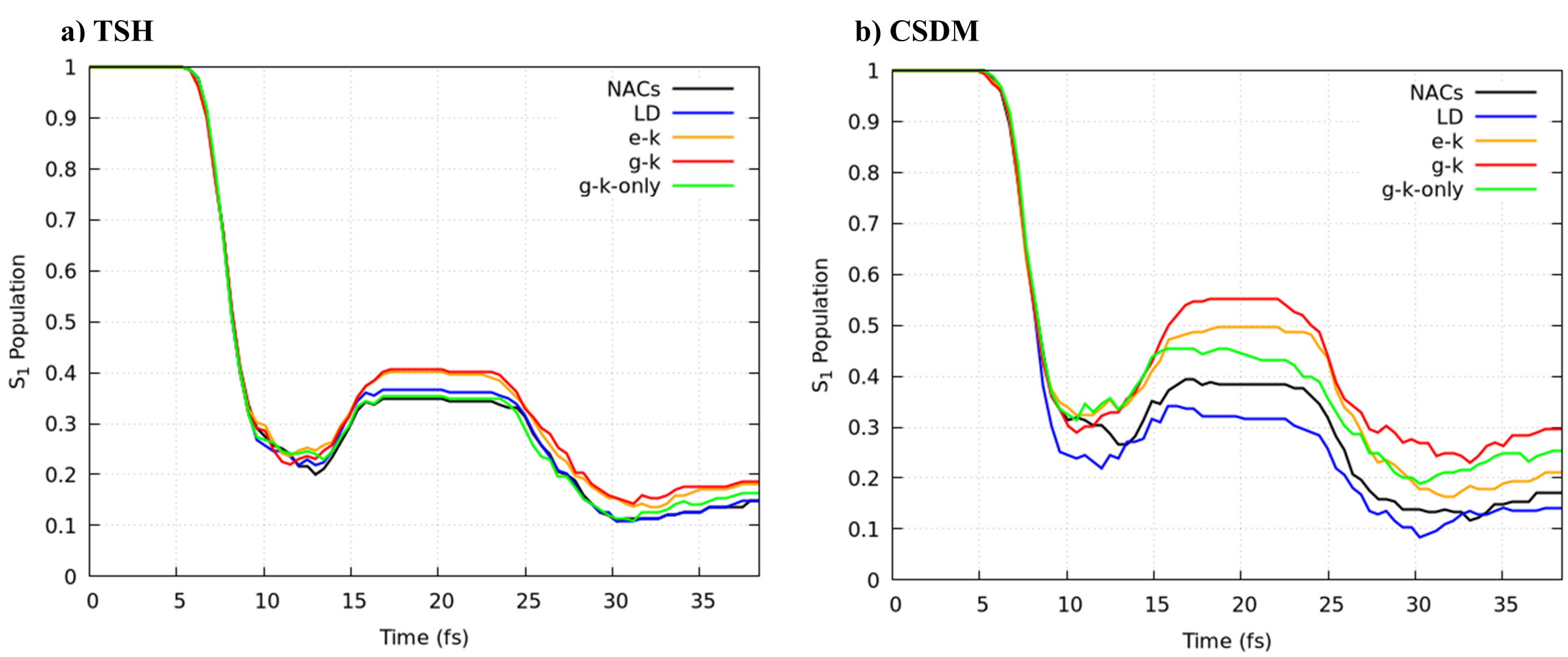

Figure S3: S_1 classical population in fulvene obtained with TSH (a) and CSDM (b) using analytic NACs (black), local diabatization (LD, in blue), e-κ (orange), g-κ (red) and g-κ-only (green).

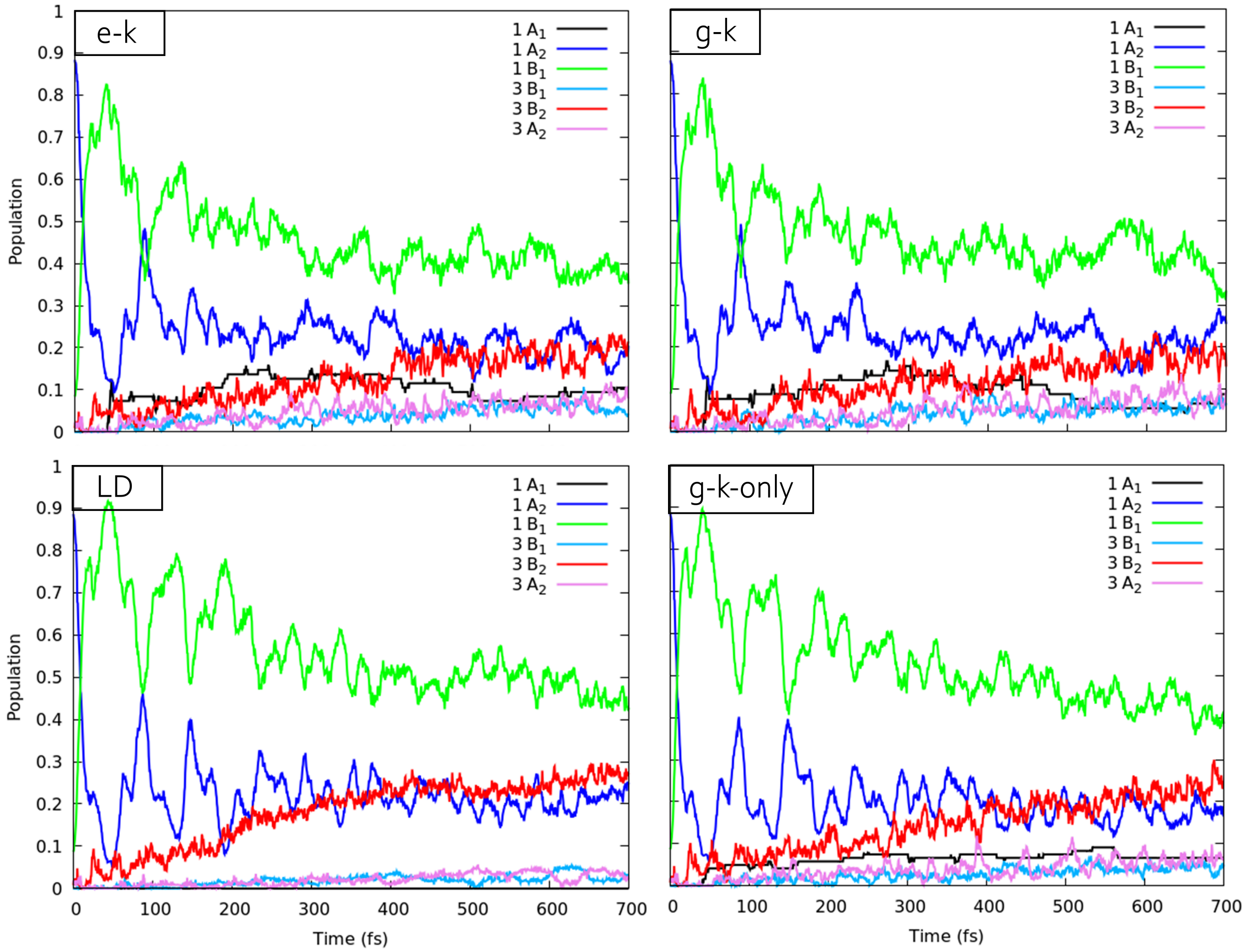

Figure S4: Classical populations for SO_2 from TSH dynamics, shown for four coupling treatments: e-κ (top left), g-κ (top right), local diabatization (LD, bottom left), and g-κ-only (bottom right). Line colors indicate diabatic states as labeled in each panel.

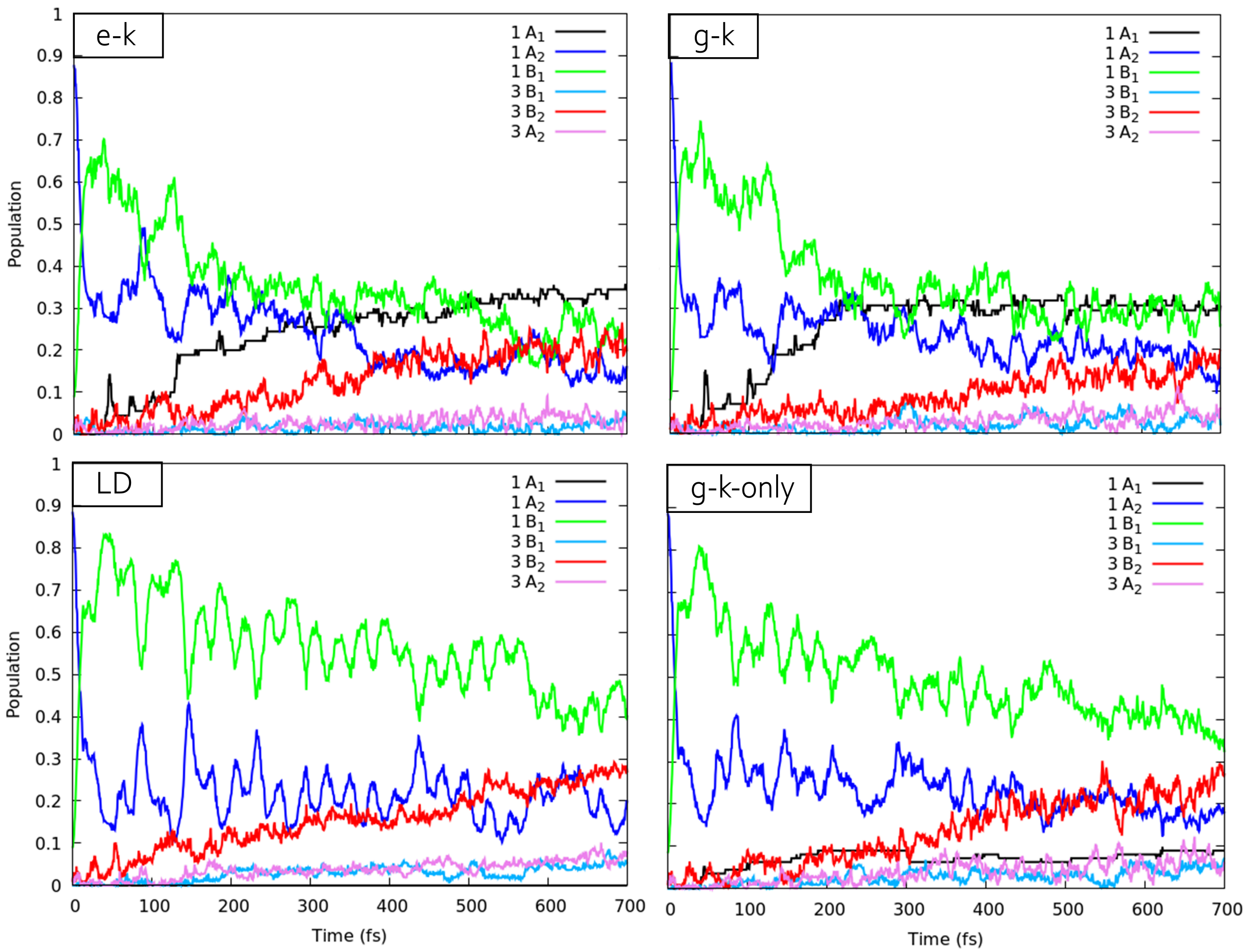

Figure S5: Classical populations for SO_2 from CSDM dynamics, shown for four coupling treatments: e-κ (top left), g-κ (top right), local diabatization (LD, bottom left), and g-κ-only (bottom right). Line colors indicate diabatic states as labeled in each panel.

2 Number of $S_2 \rightarrow S_1$ and $S_1 \rightarrow S_2$ hops in DMABN

Table S1: Time-binned number of accepted $S_2 \rightarrow S_1$ and $S_1 \rightarrow S_2$ hops in DMABN for TSH. Counts are reported in 30 fs time windows.

	$S_1 \rightarrow S_2$				$S_2 \rightarrow S_1$			
Time interval (fs)	LD	e-κ	g-κ	g-κ-only	LD	e-κ	g-κ	g-κ-only
0–30	33	28	28	23	105	104	100	79
30–60	37	59	53	22	45	44	53	36
60–90	18	37	37	18	26	55	40	20
90–120	24	45	48	16	24	40	42	17
120–150	25	42	36	21	24	45	50	17
150–180	35	45	43	19	33	39	36	22

Table S2: Time-binned number of $S_2 \rightarrow S_1$ and $S_1 \rightarrow S_2$ pointer-state switches in DMABN for CSDM. Counts are reported in 30 fs time windows.

	$S_1 \rightarrow S_2$				$S_2 \rightarrow S_1$			
Time interval (fs)	LD	e-κ	g-κ	g-κ-only	LD	e-κ	g-κ	g-κ-only
0–30	25	29	29	27	106	94	100	83
30–60	38	38	47	26	36	39	42	31
60–90	21	34	45	21	34	39	50	27
90–120	37	41	42	17	28	43	47	18
120–150	23	33	36	12	28	28	41	14
150–180	24	45	54	18	27	47	43	17

3 Single-trajectory TDCs

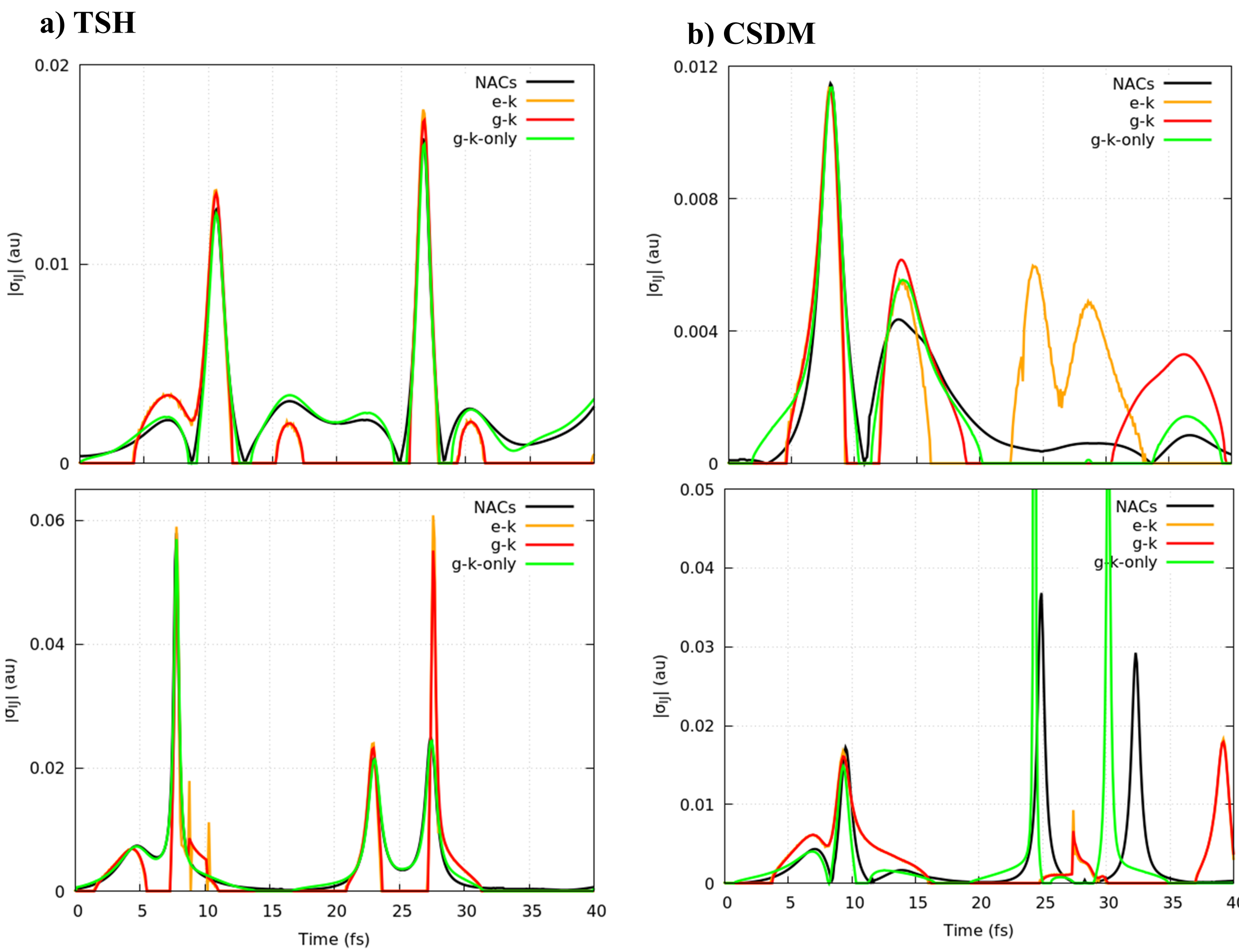

Figure S6: Magnitude of the time derivative coupling $|\sigma_{01}(t)|$ for two representative trajectories in fulvene for TSH (a) and CSDM (b) obtained from analytic NACs (black) and from curvature-driven schemes (orange: e-κ; red: g-κ; green: g-κ-only).

4 Reference MCTDH/LVC dynamics for SO_2

The MCTDH population dynamics obtained with the SO_2 LVC model and used as the reference for the comparisons in the main text are shown in Figure S7. The reference results are taken from Ref.[S1]

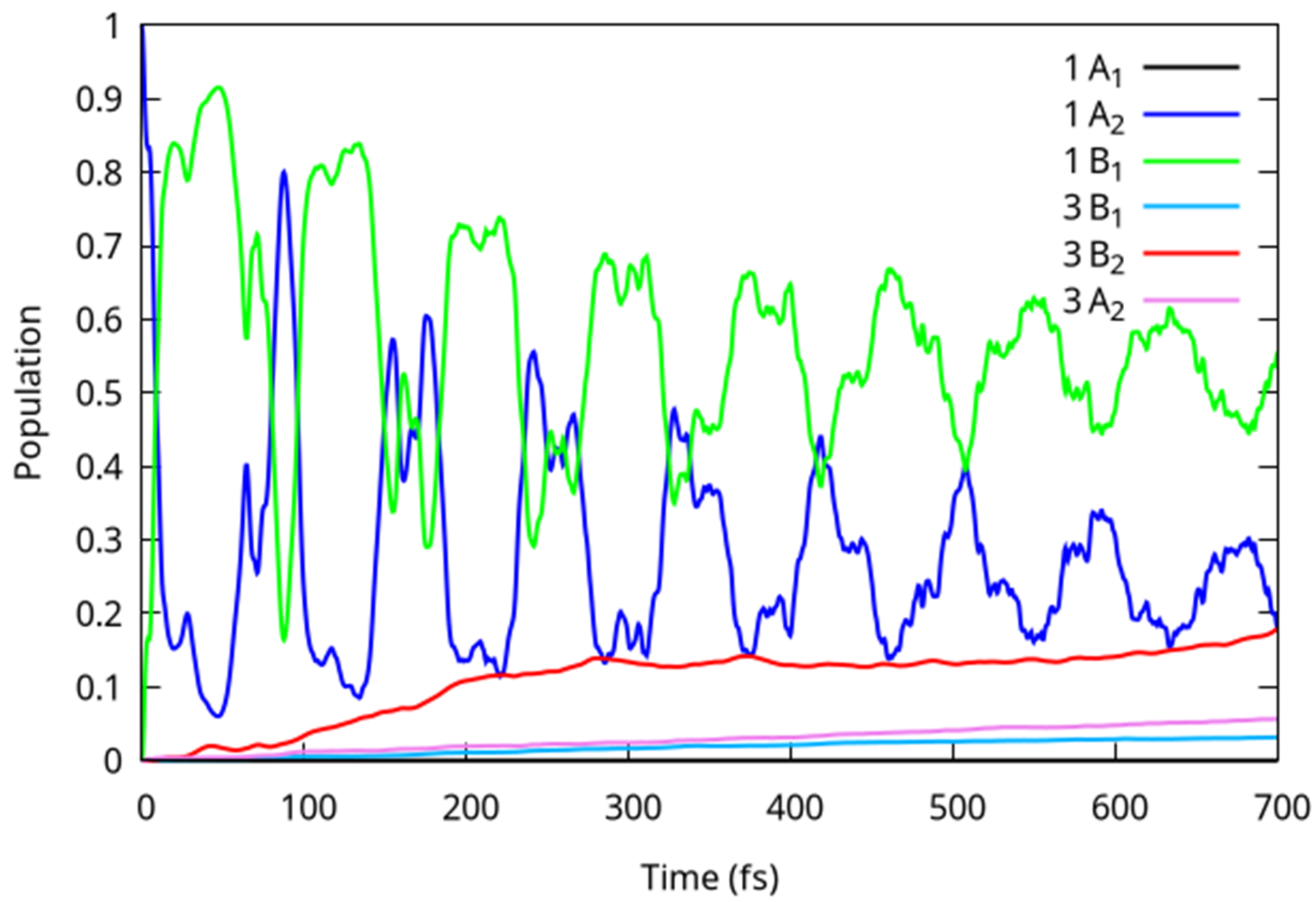

Figure S7: Reference MCTDH population dynamics for the SO_2 LVC model, taken from Ref.[S1]

5 MCH population for SO_2

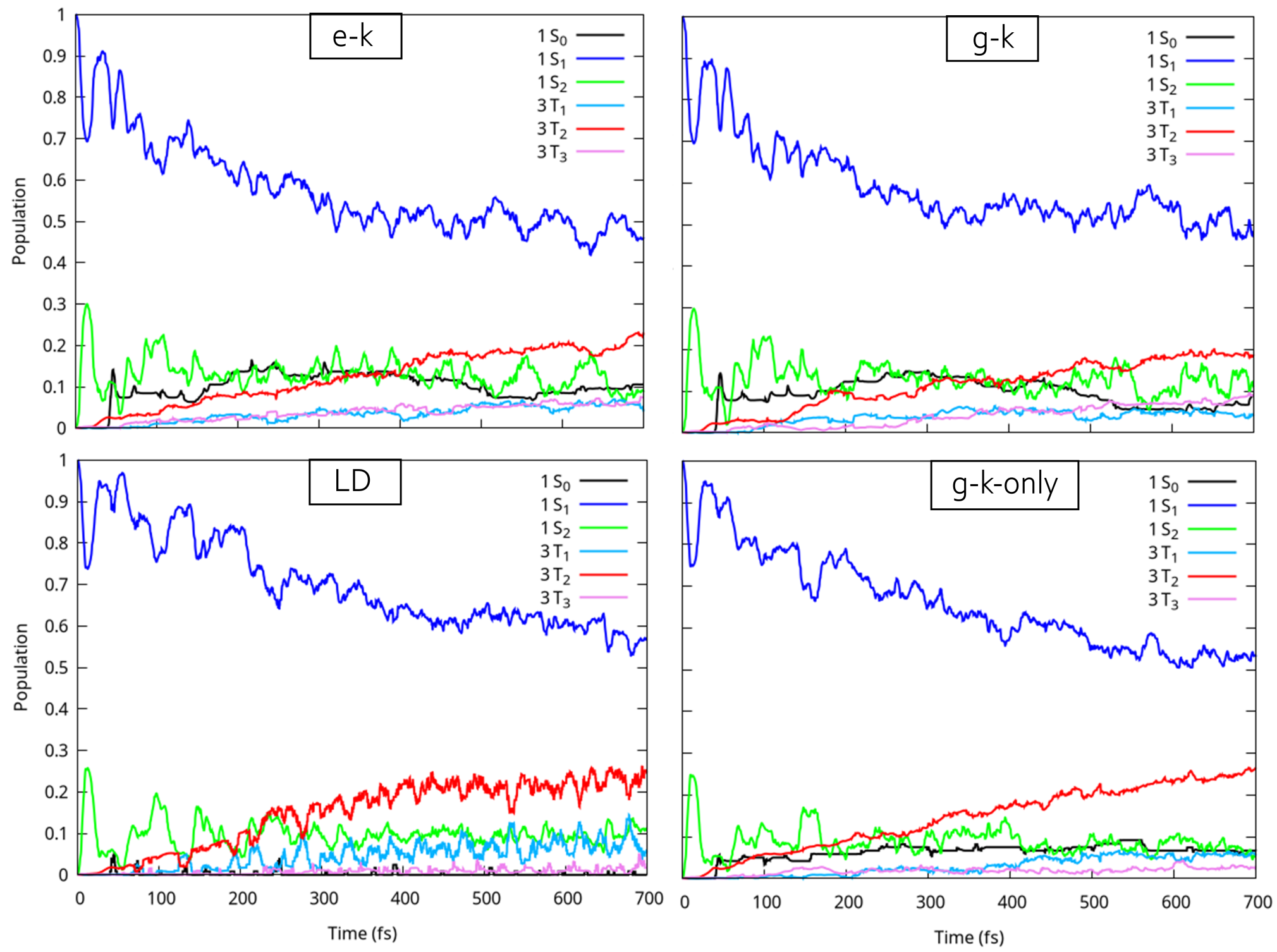

Figure S8: SO_2 MCH-state populations obtained from the TSH dynamics using e-κ, g-κ, LD, and g-κ-only.

References

(S1) Plasser, F.; Gómez, S.; Menger, M. F. S. J.; Mai, S.; González, L. Highly efficient surface hopping dynamics using a linear vibronic coupling model. *Physical Chemistry Chemical Physics* **2019**, *21*, 57–69.

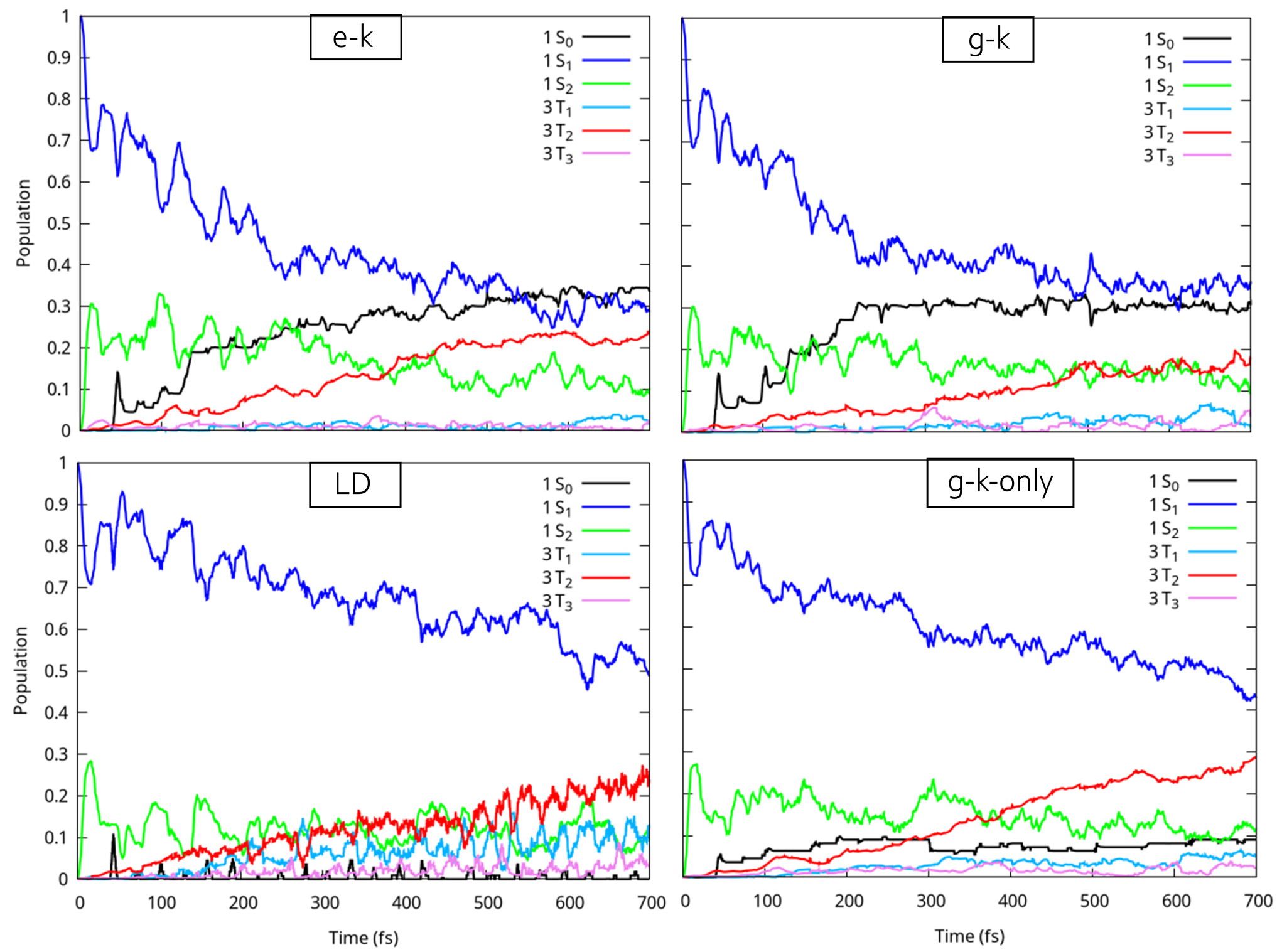

Figure S9: SO_2 MCH-state populations obtained from the CSDM dynamics using e-κ, g-κ, LD, and g-κ-only.
